# Facile and scalable disposable sensor based on laser engraved graphene for electrochemical detection of glucose

**DOI:** 10.1038/srep27975

**Published:** 2016-06-16

**Authors:** Farshad Tehrani, Behzad Bavarian

**Affiliations:** 1 Keck’s Advanced Materials Laboratory, Manufacturing Systems Engineering & Management, California State University of Northridge, Northridge, California, United States of America; 2 Corrosion Research Laboratory, Manufacturing Systems Engineering & Management, California State University of Northridge, Northridge, California, United States of America

## Abstract

A novel and highly sensitive disposable glucose sensor strip was developed using direct laser engraved graphene (DLEG) decorated with pulse deposited copper nanocubes (CuNCs). The high reproducibility (96.8%), stability (97.4%) and low cost demonstrated by this 3-step fabrication method indicates that it could be used for high volume manufacturing of disposable glucose strips. The fabrication method also allows for a high degree of flexibility, allowing for control of the electrode size, design, and functionalization method. Additionally, the excellent selectivity and sensitivity (4,532.2 μA/mM.cm^2^), low detection limit (250 nM), and suitable linear range of 25 μM–4 mM, suggests that these sensors may be a great potential platform for glucose detection within the physiological range for tear, saliva, and/or sweat.

Worldwide, over 387 million people suffer from diabetes mellitus^1^. Diabetes is a medical condition in which patients experience glucose concentration deviated from the normal range of 80–120 mg/dL (4.4–6.6 mM). According to the international diabetes federation (IDF), every 7 seconds, one person dies due to the health complications associated to diabetes, making it one of the leading causes for death in the world. In addition to death, the complications associated with diabetes increase the risk of disability and health issues of patients, including, heart disease, blindness, and kidney failure. However, these complications can be significantly reduced through strict self-monitoring of blood glucose levels. Accordingly, millions of diabetics test their blood glucose levels daily, making glucose not only the most commonly tested analyte. Because of this, glucose biosensors to account for nearly 85% of the entire biosensor market. It’s no surprise that, by as early as 2008, the market for self-monitoring blood glucose sensors (SMBG) surpassed $12 billion^2^,^3^.

Since the invention of the first glucose enzyme electrodes by Clark and Lyons^4^, there’s been tremendous amount of research in developing enzyme based amperometric biosensors, which typically rely on glucose oxidase (GOx) for detection of glucose in the blood stream^5^,^6^,^7^,^8^,^9^,^10^. The majority of SMBG is done by testing blood samples on disposable screen-printed enzyme electrode strips. This method of monitoring has two fundamental problems: first, the painful finger pricking process that may urge patients to skip their daily blood sample testing, and second, the use of a fragile and expensive GOx enzyme. It has been shown that there is a strong correlation between the glucose levels found in human blood with that of tears^11^,^12^, saliva^13^, and sweat^14^, which allows for the use of one of these alternative for actual blood glucose level detection. The average production rate of tears is 0.5–2.2 μl/min^11^. In healthy patients, tears contain a range of ≈3.6–72.2 mg/dl glucose. However, in patients suffering from diabetes, this range is typically ≈16.6–56.6 mg/dl^15^. Similarly, sweat glucose properly harvested to prevent contamination from other sources on the skin’s surface can accurately reflect the blood glucose levels of patients^14^. It has also been shown that salivary glucose has a strong correlation to fasting blood glucose levels (P < 0.05)^13^,^16^. Therefore, there is a high demand for the commercialization of an inexpensive, non-enzymatic glucose sensor sensitive enough to effectively use on the alternative body fluids of tear, sweat, or saliva.

Graphene, a two dimensional material with great surface area and superior electrocatalytic properties, has attracted the attention of scientists in a wide variety of fields, including bio-sensing applications^17^,^18^,^19^,^20^,^21^,^22^,^23^,^24^,^25^,^26^,^27^,^28^,^29^. While tremendous improvements in understanding the properties of graphene have been made since its discovery by A. K. Geim, & K. S. Novoselov^30^,^31^, there remain challenges in the applications and scalability of production at low cost for this wonder material. Production methods for graphene vary, including mechanical and chemical exfoliation^32^,^33^, chemical vapor deposition (CVD)^34^, and thermal reduction. Generally, thermal reduction of graphene has the advantage of low cost, easy processing, and the possibility of customizable patterning with laser reduction^35^,^36^. One example for a useful application of graphene using an inexpensive and scalable production method is the light scribing of the graphite oxide coated on a compact disc (CD) into graphene-based electrodes for development of supercapacitors, developed by El-kady and Kaner^35^. Another example of a simple and scalable method, developed by Lin and his colleagues, is the irritation of a commercial Polyimide film using an infrared CO_2_ laser for production of highly efficient supercapacitors. In this study, we’ve used a method similar to that presented by Lin *et al*. utilizing a different laser engraving machine for the fabrication of the sensor electrodes. The graphene electrodes formed by the direct laser engraving process is termed as direct laser engraved graphene (DLEG), Fig. 1. The DLEG working electrode is then functionalized for the purpose of sensing glucose. There is a high level of versatility in choosing the design and size of the DLEG electrodes. Additionally, the scalability of the fabrication method for production of DLEG electrodes along with the inexpensive raw materials used for development of the highly sensitive sensor platform is another highlight of this application.

Copper nanoparticles are widely used as an electrode modifier, possessing great catalytic functionality and synergy with other nano structures, such as carbon nanotubes (CNT) and graphene. For glucose sensors, these copper nanoparticles are typically coated on the surface of glassy carbon-graphene substrate^37^,^38^,^39^,^40^. In this study, nano cubic structured coppers (CuNCs) were electrochemically deposited on DLEG using a pulse electrodeposition technique. This worked as a seamless process for generation of highly sensitive glucose sensor strips capable of detecting low glucose levels found in human tear, saliva, and sweat. To the best of our knowledge, this is the first instance of pulse electrodeposition of cubic copper nano particles.

## Results and Discussion

The laser engraved Kapton was characterized by Raman spectroscopy, field emission scanning electron microscopy (FESEM), energy dispersive x-ray spectroscopy (EDS), and x-ray photoelectron spectroscopy (XPS). As was seen in Lin’s research^41^, the Raman spectrum (Fig. 2i) showed three distinguished peaks for the DLEG. However, no clear peaks were seen for the none-engraved Polyimide within the range of 1200–3000 cm^−1^. The first order D peak, (roughly at 1,350 cm^−1^) is reflected by either sp^2^ bonds between atomic carbons or lattice defects. The second peak, a first order G peak (roughly at 1,580 cm^−1^) is common in both graphene and bulk graphite. The third peak, which is the second order D peak (also known as the 2D peak), with a singular and sharp shape, is unique to 2 dimensional graphite with randomly stacked graphene sheets in the c axis^41^,^42^ and was observed at roughly 2,700 cm^−1^. Additionally, the 2D peak for the bulk graphite is frequently observed with two components of 2D_1_ and 2D_2_, and is significantly different in shape and intensity from that of DLEG^42^,^43^,^44^. Ultimately, the intensity ratio of the D/G confirms a great extent of graphene formation^41^ during the laser engraving process. Furthermore, the clear difference between Raman spectrum of the DLEG with that of glassy carbon eliminates the possibility of glassy carbon being produced as the primary material in the engraving process. The XPS spectrum of the bare DLEG (Fig. 2h,j) shows dominant C-C peaks at 278.8 eV, with highly repressed C-O, C-N, and C=O peaks. The high resolution XPS on the DLEG (Fig. 2j) agrees well with the Raman results, and is suitably matched with that of graphene. This suggests the presence of sp^2^ carbons in graphene formed in DLEG during the engraving process^41^,^45^.

DLEG units with a lateral length of roughly 20 μm (height of 25 μm from the tilted FESEM image, not shown) is apparent in the FESEM image of the sensor’s electrode, seen in Fig. 2a. The FESEM of the bare DLEG (Fig. 2b) revealed interconnected nano-sheets that are thin enough to be transparent to the electrons scattered from the scanning electron microscope. Additionally, STEM images on the DLEG flakes suspended on top of the carbon grid were taken using the FESEM machine in a STEM mode (See the section for CuNCs electrodeposition), showing thin overlapped graphene flakes with different number of layers. A uniform, high density deposition of copper nano-cubes (particle size < 30 nm) was observed in the FESEM images of the modified electrode (Fig. 2c,d). The EDS from two different spots of the DLEG-CuNCs (Fig. 2g insets) revealed a 9.95–15.64 weight % copper, 83.39–88.39 weight % of C, and 0.97–1.66 weight % of O. XPS spectrum of the bare DLEG (Fig. 2h) further confirmed the EDS results by showing a high intensity C1s peak at a binding energy of 285.00 eV (with 92.72% carbon mass concentration), two relatively low intensity peaks of N1s at a binding energy of 400.00 eV (with 2.47% nitrogen mass concentration), and O1s at a binding energy of 533.00 eV (with 4.81% oxygen mass concentration). The relatively low mass concentration of nitrogen and oxygen on the DLEG is due to their evaporation and liberation from the Kapton, suggesting a high level of graphitization during the laser irritation of Kapton with a known composition of 70.5%, 22.5%, and 7.0% for C, O, and N respectively^41^. The XPS spectrum of the DLEG-CuNCs composite at 60–480 seconds of Ar-ion etching at 4 KV (0.7 current) shows a rather large Cu2p peak at a binding energy of 933.00 eV (in the range of 9.80–49.63% copper mass concentration depending on the depth), indicating that the deposition of is primarily metallic copper (Cu) with secondary copper oxide species such as CuO (Cu (I)) and Cu_2_O (Cu (II)) during the pulse electroplating^45^.

**Figure 1:**
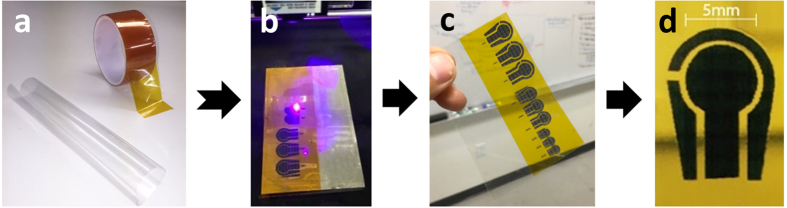
Fabrication process. Taping Kapton tape on a thin sheet of PVC **(a)**. Direct laser reduction of the Kapton tape to graphene forming sensor electrodes **(b,c)**. A DREG 3-electrode platform, prepared for further modification **(d)**.

**Figure 2:**
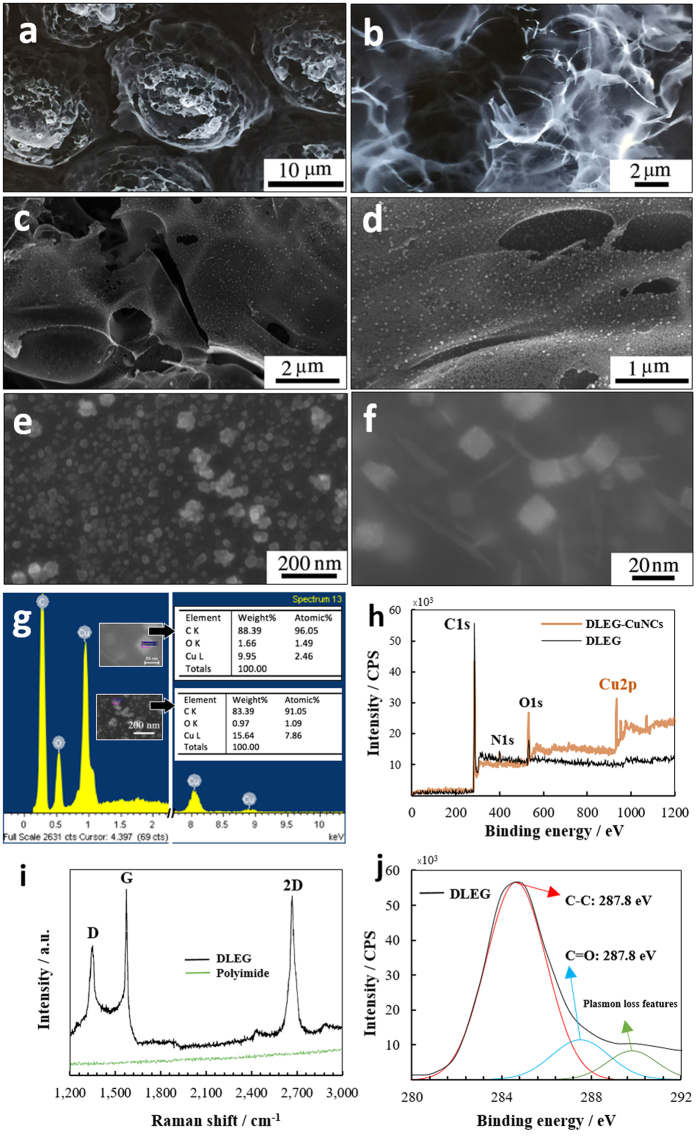
Materials Characterization. (**a,b)** FESEM images of bare DLEG, (**c–e)** and uniformly deposited CuNCs at different magnifications with a high population density on the DLEG sheets. (**f)** High magnification FESEM showing the CuNCs with a cubic structure. (**g)** EDS results of the graphene-copper nanocomposite from two different spots on the sample (spectrum 1 & 13) that indicates the presence of 83.39–88.39 & 9.95–15.64 weight percent of carbon and copper respectively. (**h)** XPS spectrum on DLEG, and DLEG-CuNCs showing a prominent C1s peak and small N1s & O1s peaks for the bare DLEG as well as the extra Cu2p peak that indicates the presence of metallic copper and Cu (I) oxide and Cu (II) oxide on the modified electrode. **(i)** Raman spectrum of the polyimide vs. the DLEG demonstrating prominent D, G, and 2D peaks that suggest a large degree of graphene formation during the engraving process on the polyimide. **j)** High-resolution XPS analysis for the C1s peak showing a dominant C1s and a suppressed C=O peak which is in a great agreement with that of graphene.

**Figure 3:**
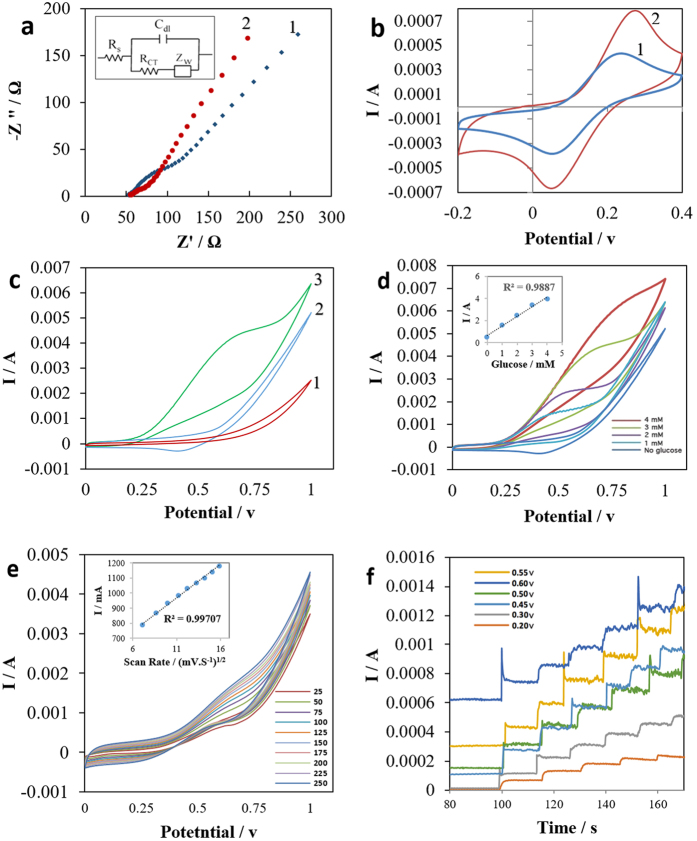
Electrochemical Characterization. (**a)** Nyquist plot of the bare graphene electrode (1), and modified CuNCs-graphene electrode (2), in a 100 mM KCl solution containing 2 mM K_3_[Fe(CN)_6_] + 2 mM K_4_[Fe(CN)_6_] (1:1). Inset is the equivalent Randle circuit. (**b)** CV curves of DLEG (1), as compared to CuNCs-DLEG (2) in 100 mM KCl solution containing 2mM K_3_[Fe(CN)_6_] + 2mM K_4_[Fe(CN)_6_] (1:1). (**c)** CV curves of the Bare DLEG (1) and modified CuNCs-DLEG electrodes in the absence (2) and presence (3) of 3 mM Glucose in 0.1 M NaOH solution. (**d)** CV curves of the modified CuNCs-DLEG in the presence of 0, 1, 2, 3, and 4 mM Glucose in a 0.1 M NaOH solution with constant scan rate of 100 mV/s. The inset plot is the linear correlation between the glucose concentration and the output signal. (**e)** CV curves of CuNCs-DLEG in 0.1 M NaOH with 2 mM Glucose at different scan rates ranging from 25 mV/s to 250mV/s, with a step size of 25 mV/s. The inset is a plot of the peak current vs. square root of the scan rate at an applied potential of 0.55 v. (**f)** Optimization of applied potential using amperometric current response of CuNCs-DLEG at different potentials in 0.1 M NaOH with successive addition of 0.1 mM glucose.

EIS testing was conducted in order to evaluate the charge transfer behavior of the DLEG and DLEG-CuNCs electrodes. The results are shown in the form of Nyquist complex plots (Fig. 3a). Fitting the Nyquist plots to their equivalent Randle’s circuit model (Fig. 3 inset) gave a calculated solution impedance of R_s_ = 50 Ω, and an impedance to the charge transfer rate at the surface of the bare DLEG equal to Rct = 65 Ω. The solution impedance, R_s_, that appeared in the high potential frequency region of the Nyquist plot is directly proportional to the concentration of the free ions in the solution. The low impedance to the charge transfer at the surface of the bare DLEG electrode, which appeared at lower potential frequencies in the Nyquist plot, is attributed to both the high conductivity and the large surface area of the porous DLEG. Comparing the Nyquist plots of the bare and modified electrode (Figs 2 and 3a-1) shows that the impedance to the charge transfer rate is reduced to R_ct_ = 21 Ω from the DLEG to DLEG-CuNCs. The observed increase in the charge transfer rate from DLEG to DLEG-CuNCs can be attributed to the increase in the electrode’s surface area, caused by the formation of highly conductive nano cubic particles of copper. The increase in the charge transfer rate of the bare electrode after deposition of CuNCs was further confirmed via Cyclic Voltammetry (CV) experimentation on the same electrodes, using a 100 mM KCl solution containing 2 mM K_3_[Fe(CN)_6_] + 2 mM K_4_[Fe(CN)_6_] (Fig. 3b). The CV of the DLEG showed a cathodic peak current of i_pc_ =  434 μA (Fig. 3b, curve #1), which, after the electrode’s modification to DLEG-CuNCs, was increased to i_pc_ = 782 μA (Fig. 3b curve #2).

The Cyclic Voltammetry (CV) experimentation on bare and modified samples were conducted in a 100 mM NaOH solution, tested in both the absence and presence of a 3 mM glucose, with a scan rate of 100 mV/s from 0 to +1 V. The results are demonstrated in Fig. 3c. By comparing the CV behavior of the DLEG (Fig. 3c, curve # 1) to CuNCs-DLEG (Fig. 3c, curve # 2), in the absence of the glucose, an increase in the charge transfer rate of the bare electrode to the modified electrode was observed. After the addition of 3 mM glucose for the bare DLEG system, no redox peaks were observed. However, in the presence of 3 mM glucose, the CuNCs-DLEG electrode (Fig. 3c, curve # 3) demonstrated a rapid increase in the current, starting at around 200 mV, followed by a clear, irreversible shoulder oxidation peak at around 600 mV. There was a corresponding current density of roughly 4.5 mA. The high peak potential in the electrode decorated with CuNCs indicates the strong electrocatalytic feature of the CuNCs toward direct oxidation of the glucose in the basic solution. In order to further characterize the sensitivity of the CuNCs-DLEG electrode, CV testing of the modified after the addition of 1–4 mM glucose in 100 mM NaOH solution were performed on the modified electrode. As a result, a linear correlation between the glucose concentration and the output current at the CV’s oxidation peak was established (Fig. 3d). The linearity that was established conveys the workability of the working electrode towards glucose sensing.

The effect of scan rate on the oxidation of glucose was investigated in a 100 mM NaOH solution with presence of 2 mM glucose by using the CuNCs-DLEG electrode. Multiple CV tests were carried out at scan rates of 25–250 mV/s with a scan steps of 25 mV/s (Fig. 3e). The anodic peak (I_p_) vs. square root of the scan rate shows a highly linear correlation with correlation constant R^2^ = 0.997 and I_p_ = 0.0227SR+11 (Fig. 3e, inset plot). This confirms that the electrochemical oxidation kinetics in the sensor were controlled by the adsorption of the glucose molecules at the surface of the working electrode. Furthermore, CV curves with different scan rates stabilized within 3 cycles. After stabilization, the CV curves consistently repeated the same pattern over the next 20 cycles, indicating the high electrochemical stability of the CuNCs-DLEG platform in the basic solution with the presence of glucose.

In order to optimize the potential for oxidation of the glucose molecules at the working electrode, multiple amperometric experiments were performed at different potentials with successive addition of 0.1 mM glucose into a 0.1 M NaOH solution. Six different potentials (0.2 V, 0.3 V, 0.45 V, 5 V, and 0.6 V) were evaluated using the same sensor. The strongest amperometric signal response (approximately 170 μA per addition of 0.1 mM glucose, with regular step-like responses) was observed at the applied potential of 0.55 V (Fig. 3f). Therefore, 0.55 V is considered to be the optimal sensing potential for the sensor.

**Figure 4:**
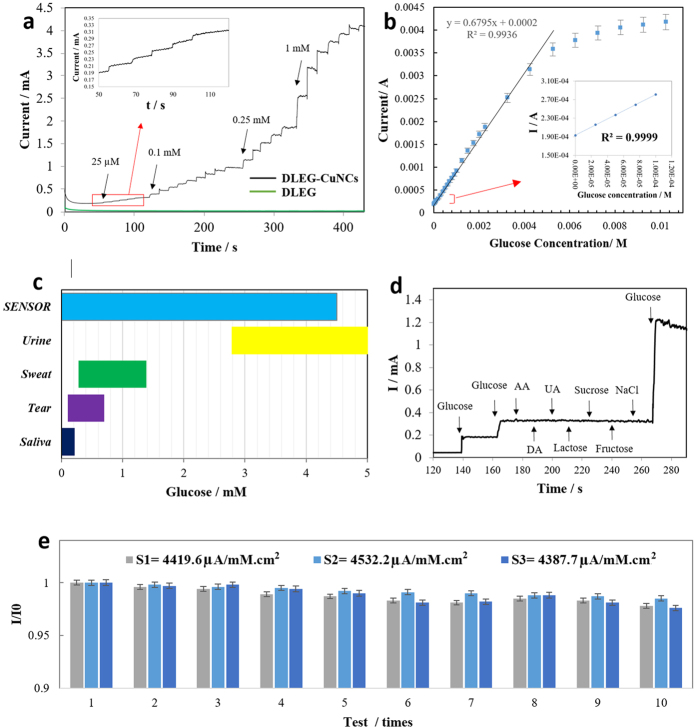
Sensor's performance. (**a)** Amperometric current response at +0.55 V with successive addition of different glucose concentrations including 25 μM (5 times, with magnified inset), 0.1 mM (9 times), 0.25 mM (5 times), and 1 mM (7 times), illustrated with the black curve. Amperometric response of the none-modified electrode (DLEG) with a negligible current response, green curve. (**b)** Calibration curve of the sensor. (**c)** The sensing linear range of the sensor as compared to physiological levels of glucose found in sweat, tear, saliva, and urine. (**d)** Selectivity experiment using amperometric current responses of sensor with successive addition of 0.25 mM glucose (twice), then 0.02 mM electroactive interfering species of AA, DA, UA, Lactose, Sucrose, Fructose, and 0.2 mM NaCl, and finally 1 mM glucose into 0.1 M NaOH at an applied potential of 0.55 V. (**e)** Reproducibility (3 sensors were compared) and stability (compared with the value of the first test) of the sensors 1, 2, and 3 stored at normal ambient conditions using 0.1 mM glucose in 100 mM NaOH at 0.55 v of applied potential, conducting repeating amperometric experiments every three days for a total of 10 times during one month.

The amperometric response of the glucose sensor (Fig. 4a) was evaluated by conducting a chronoamperometry experiment after the addition of glucose drops with concentrations of 0.25 μM, 0.1 mM, 0.25 mM, and 1 mM into the testing vial. The calibration curve (Fig. 4B) is plotted to demonstrate the current-glucose concentration correlation. The plot shows a linear behavior with R^2 ^= 0.99357 (y = 0.6795x + 0.0002), sensitivity of ≈4532.2 μA/mM.cm^2^, linear range of 0.25 μM–4 mM, detection limit of 250 nM, and a rapid amperometric response of < 3 seconds. The mechanism for the oxidation of glucose in alkaline media at the CuNCs modified electrode is believed to be as follows: CuNCs upon exposure to NaOH (the supporting electrolyte) will oxidize into CuO [Cu + 2OH^−^ → CuO + H2O + 2e^−^]. Subsequently, CuO is electrochemically oxidized to Cu(III) species, such as CuOOH and/or Cu(OH)4^−^ [CuO + OH^−^ → CuOOH or CuO + H2O + 2OH− → Cu(OH)4^−^ + e^−^]. Finally, glucose is irreversibly oxidized by the Cu(III) species, forming hydrolyzate gluconic acid [Cu(III) + glucose → gluconolactone + Cu(II)] [Gluconolactone → gluconic acid (hydrolysis)]^46^,^47^,^48^ The thermal (laser) reduction of polyimide creates a porous nano-sheet structure of graphene with an abundance of crystallographic defects and functional groups, such as −OH, C–O–C, and −COOH, which provides opportunity for catalytic applications^35^,^41^. Hence, the porous DLEG is believed to be a great substrate for electroplating of the CuNCs (as the catalyst for oxidation of glucose). Furthermore, the large surface area of the porous, double sided DLEG promotes an enhanced loading of highly reactive CuNCs and accessibility of the sensing molecule (glucose) as well as an electrochemically robust conductive DLEG in an alkaline environment. These are advantageous attributes of the DLEG-CuNCs nano-composite, making it an excellent synergic material for glucose sensing applications. Ultimately, the suitable linear range of the sensor allows for the possibility of glucose detection in tears^49^, saliva^50^, sweat^14^, and partial in urine^51^ (Fig. 4c). Table 1 shows the performance of the sensor compared with some other graphene-based sensors. In addition to the differences shown in Table 1, our sensor was fabricated on a flexible and facile plastic substrate, and can be fabricated on essentially any other substrate. The other sensors shown in the table were fabricated on costlier substrates: a highly conductive and expensive glassy carbon for one, and a silicon-silicon dioxide for the other. The seamless fabrication method and low cost of the raw materials for our sensor are some of the major advantages of our sensor when compared to the others.

The effect of the some of the electrochemically activating molecules found in the human serum (0.02 mM ascorbic acid (AA), uric acid (UA), sucrose, fructose, and lactose, as well as 0.2 mM NaCl) were investigated on the sensor by performing a chronoamperometry experiment. The results shown in Fig. 4d show no significant signal from any of the interferants when compared to the signals of the glucose droplets (0.25 mM and 1 mM). This implies that the sensor has excellent selectivity towards glucose molecules.

The reproducibility of the samples 1, 2, and 3 is measured by comparing the signal response (sensitivity) of the samples in presence of 0.1 mM glucose/0.1 M NaOH solution. The results show great reproducibility among the 3 samples (> 96.8%) with respective sensitivities of 4,419.6, 4,532.2, and 4,387.7 μA/mM.cm^2^ (Fig. 4e). The high reproducibility of the sensor, along with the potential for mass fabrication and low material cost indicates a strong potential for commercialization.

The stability of the sensor was systematically evaluated by performing multiple amperometric experiments over a 30-day period. Every three days, samples 1, 2, and 3 were tested in the presence of 0.1 mM glucose/0.1M NaOH solution. The samples were then stored in a petri dish in a typical ambient environment (25 °C). The first experiment’s first output signal, I_0_, was used to normalize the subsequent experiments in order to understand the samples’ stability (I/I_0_). From results, shown in Fig. 4e, a stability rate of >97.4% is calculated. This indicates considerably low signal loss (maximum of 1.3%) over the repetitive use of the sensor strips during the 30-day period (each sample tested 10 times). The high stability, despite the repeated use and exposure to the corrosive NaOH environment, is attributed to the high electrochemical stability of the DLEG platform.

**Figure 5:**
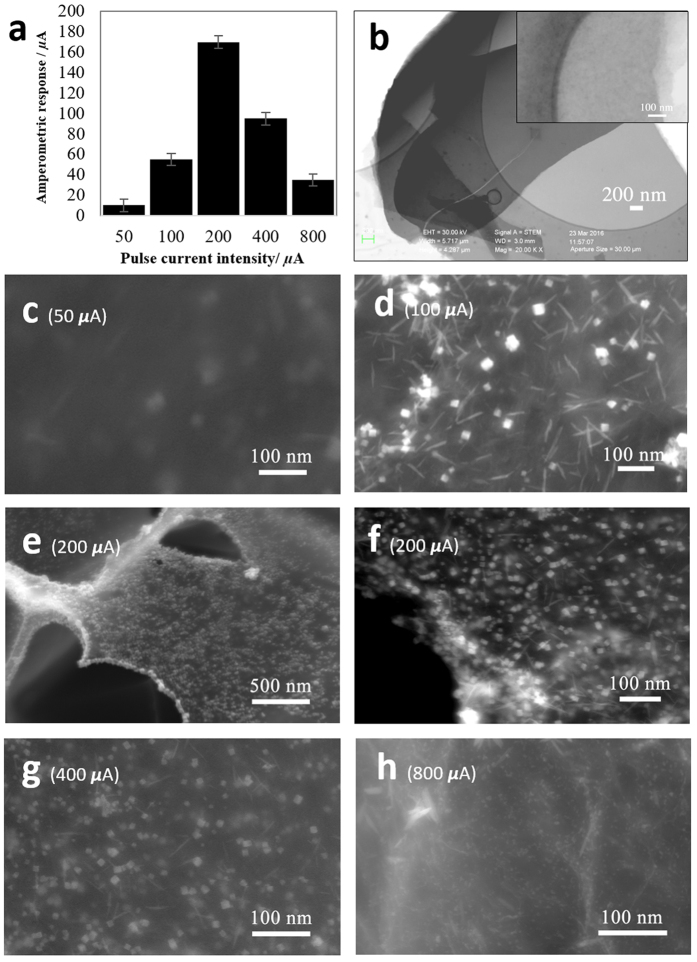
Electrodeposition optimization. (**a)** Amperometric current response of the working electrodes as a function of the electroplating pulse current intensity. (**b)** STEM image of bare DLE graphene flakes on top of the grid with a higher magnification inset before electroplating with CuNCs. (**c–h)** FESEM Images of the DLEG-CuNCs electrodes at different electroplating pulse current during the electroplating process suggesting the formation of the finest CuNCs with great uniformity and population density at 200 μA and less CuNCs population density for all the other applied current.

Pulse electrodeposition was used for deposition of the CuNCs. According to Grujicic, and Pesic^49^, the copper nucleation mechanism is a function of the solution pH, copper ionic concentration, deposition potential, temperature, and background electrolyte. Additionally, the deposition time is directly proportional to the size of the copper particles. Therefore, a greater deposition time would result into the greater growth of the copper nuclei and, subsequently, the formation of larger copper nano particles. Therefore, the size, population density, and morphology of the CuNCs can be controlled by the variables mentioned above. For our research, in order to simplify the deposition process while achieving the optimal size and population density of the CuNCs, the electrodeposition solution concentration, pH, temperature, and deposition time were held constant at the optimal values established in our previous study^52^. The pulse current intensity, the only variable introduced in the newly employed pulse electroplating technique, was manipulated in favor of electroplating optimal CuNCs. Unlike the previous research which employed a potentiostatic technique, we used a pulse electroplating technique with variable current intensities of 50 μA, 100 μA, 200 μA, 400 μA, and 800 μA for 350 cycles (squared 1:1 second). According to the chronoamperometry results obtained on all the electroplated samples, a pulse current intensity of 200 μA produces the greatest current response of ≈170 μA (Fig. 5a). As expected, the FESEM results of the samples (Fig. 5b–j) revealed that the 200 μA electroplated sample produced the finest CuNCs (<30 nm) with a great uniformity and population density (Figs 2c–f and 5d,e). For the samples electroplated at 50 μA and 800 μA (Fig. 5b,j), there are almost no observable CuNCs nuclei deposited on the graphene sheet. However, for the samples with 100 μA, and 400 μA (Fig. 5c,f) of applied pulse current, CuNCs are formed on the graphene substrate; their population density and particle size are not as optimal as the 200 μA electroplated sample, and do not produce enough catalytic activity. Therefore, it is confirmed that the larger surface area provided by the smaller and denser CuNCs on the DLEG surface increases the glucose-CuNCs interaction and, subsequently, the chemical reaction leading to the oxidation of glucose. Hence, an optimal electroplating condition for the pulse electroplating technique is believed to be achieved at 350 cycles of an applied current intensity of 200 μA in an electroplating solution of 5 mM CuSO_4_ + 50 mM Na_2_SO_4_.

## Methods

### Reagent and Materials

K_3_[FE(CN)_6_] and K_4_[Fe(CN)_6_], CuSO_4_, Na_2_SO_4_, D-glucose, Ascorbic Acid (AA), and Uric Acid (UA) were purchased from Sigma Aldrich Co. (USA). All the other reagents were of analytical grade and used without further purification. Solutions were made using Millipore water (≈18 MΩ). Kapton (polyimide) tape with a film thickness of ≈30.4 μm and a width of ≈50 mm was purchased from McMaster-Carr co. (USA).

### Synthesis of graphene electrodes

A 3-electrode system was designed with CorelDraw Graphics Suit ver. X8 and engraved to DLEG-based electrodes (working, auxiliary, and reference) in the following steps. First, a piece of Kapton tape (Polyimide) was taped onto a PVC (polyvinyl chloride) transparent substrate and was placed into a laser engraving machine. Next, the designed pattern made in a graphic software is engraved to the surface of the Kapton tape to form highly conductive DLEG electrodes seen in Fig. 1. The sheet resistance of the DLEG, which has a direct impact on the charge transfer rate of the electrodes and, ultimately, the sensitivity of the sensor, was optimized by adjusting the laser power intensity (80% of the machines maximum power ≈ 400mW), beam-substrate incident focus size (≈ 30 μA), and distance between the laser beam and the Kapton substrate (≈ 13 cm). DLEG samples with an average sheet resistance (R_s_) of 14.3 Ω/square were produced and used as the sensing platform. All the three DLEG electrodes (Fig. 1d), including the working electrode (diameter = 5 mm), were padded using copper tapes. A silver paste was used to improve the connection over repeated use of the sensor. The laser engraving process on the Kapton tape was carried out in the ambient conditions (in the air at 25^°^C) by using a commercially available DYI 500 mW violet laser engraving machine with a laser wavelength of 400–450 nm pulse duration of ≈ 50 milliseconds, and a beam size of ≈ 1.3 mm. The duration for fabricating a complete DLEG sensor platform was less than 6 minutes.

### Copper nanocubes electrodepositing

The DLEG working electrode was modified by using pulse electrochemical deposition of Copper Nano Cubes (CuNCs) at an optimized pulse current intensity of 200 μA for 350 cycles (squared 1:1 second). The electroplating was conducted on the same 3-electrode system prepared in the previous steps while in an electroplating solution of CuSo_4_ (5 mM) and Na_2_So_4_ (50 mM). (see the last paragraph in the “Results and Discussion” section for more details).

### Characterization

A Zeiss Ultra 55 Field Emission Scanning Electron Microscope (FESEM) and a JEOL JSM-6480LV SEM were used for the study of the electrodes’ nanostructure. X-ray Photoelectron spectroscopy was performed on samples as received and after 60–480 seconds of Argon-ion etching at 4KV (0.7 μA of current) using an Oxford AXIS ULTRA instrument. Raman spectrum of the DLEG sample for confirming the formation of graphene from the polyimide during the engraving process was obtained by using a DXR2 Raman spectrometer from Thermo Scientific with a laser power of 5 mW, at a laser excitation of 514.5 nm, and 2 seconds of integration time. Energy-Dispersive X-ray Spectroscopy (EDS) of the sensor was implemented by using X-Mass from Oxford Instruments. No additional processing steps were performed on the sensors before the characterizations.

### Electrochemical experiments

All electrochemical measurements and corresponding data fittings were carried out on a Gamry Instruments Ver. 4.35, 2005. A 3-electrode system, all made of DLEG, was used with the reference electrode coated with a Ag/AgCl Paste. Therefore, all the potentials are reported with respect to a Ag/AgCl reference electrode with a standard potential of 230 mV ± 10 mV (vs. SHE). Electrochemical Impedance Spectroscopy (EIS) experiments were carried out in 100 mM KCl solution containing 2 mM K_3_[Fe(CN)_6_] +2 mM K_4_[Fe(CN)_6_] (1:1) in the frequency range of 100 KHz- 0.1 Hz. The EIS results were plotted in the form of complex plane diagrams (Nyquist plots) and were fitted to the equivalent Randles circuit. Cyclic Voltammetry (CV) experiments were performed in 0.1 M NaOH aqueous solution both with and without glucose. The chronoamperometry and CV experiments were performed under a constant stirring condition by using a magnetic stirrer bar with a length and thickness of 8 and 2.5 mm. A pipette with a droplet size of approximately 24 μL/drop was used for successive addition of concentrated glucose solution into the testing vial. All the experiments were performed at the room temperature. All reagents were analytical grade and ultra-pure water (≈18 MΩ) was used in all of the electrochemical experiments.

### Limitations

The major limitations associated with our study is related to the fact that all the experiments were implemented in an *in-vitro* setup. Hence, further study of the sensor performance using real samples of human tears, saliva, sweat, and partial urine would be required to validate the findings in this study for real life application.

### Future work

In addition to what has been discussed, the following features of the DLEG electrode can create an opportunity for future developments toward commercialization of the sensor. First, compatibility of the CuNCs-DLEG fabrication method with conventional screen printing technology which would allow the mass production of inexpensive disposable test strip platforms and tattoo-like wearable patches. Second, applicability of the variety of modification and functionalization techniques on the sensitive DLEG working electrode, which would offer fabrication of a wide range of bio-sensors; Therefore, bringing about development of novel sensing strips for detection of other important molecules in the body fluids such as AA, UA, cholesterol, alcohol and etc. Third, the feasibility of micro-scale fabrication of the flexible DLEG electrodes, which makes it possible to integrate the DLEG electrodes possible in disposable contact lenses for glucose sensing applications.

## Conclusion

We developed a sensitive, enzyme-less glucose sensor using inexpensive fabrication method and materials. Excellent sensitivity, specific linear range, as well as a low detection limit are among the strong attributes of the glucose sensor. High stability of the DLEG-CuNCs electrodes along with a high reproducibility rate, and the feasibility for in-scale production of the electrodes allow for commercialization of the sensor. Additionally, the laser engraved graphene electrode developed in this study offers a novel approach for development of an inexpensive, but a highly sensitive platform for electrochemical bio-sensing applications.

## Additional Information

**How to cite this article**: Tehrani, F. and Bavarian, B. Facile and scalable disposable sensor based on laser engraved graphene for electrochemical detection of glucose. *Sci. Rep*. **6**, 27975; doi: 10.1038/srep27975 (2016).

## Figures and Tables

**Table 1:** Comparison of several sensors performance.

Sensor	RT (s)	Potential (v)	Detection Limit (μM)	Linear Range (mM)	Sensitivity (μAmM^−1^cm^−2^)	Reference
CuNCs-DLEG	<3	0.55 (vs. Ag/AgCl)	0.25	0.025–4.5	4532	This work
GC/CoNSs/CHIT-RGO	–	0.45 (vs. SCE)	10	0.015–6.95	1921	53
GC/Cu Nanocubes/Graphene	–	0.55 (vs. Ag/AgCl)	1	Up to 7.5	1096	54
GC/CuNPs/Graphene	<2	0.50 (vs. Ag/AgCl)	0.50	Up to 4.5	–	55
SPE /Pt-Cuo/rGO	–	0.60 (vs. Ag/AgCl)	0.01	0.0005–12	3577	56
Si-SiO_2_/Ti/Pt/GNP/PEDOT-GOx	–	–	0.03	0.01–50	≈14	57,58

## References

[b1] International Diabeties Federation (IDT) - Facts and Figures about Diabeties- (2014), Available at: http://www.idf.org/worlddiabetesday/toolkit/gp/facts-figures (Accesses: 3rd Augest 2015).

[b2] Wang J. Electrochemical Glucose Biosensors. Am. Chem. Soc. 108(2), 814–825 (2008). 10.1021/cr068123a 18154363

[b3] Hughes M. D. The Business of Self-Monitoring of Blood Glucose: A Market Profile. J Diabetes Sci Technol (Online) 3(5), 1219–1223 (2009). 10.1177/193229680900300530 PMC2769893 20144440

[b4] Clark Champ Jr. Lyons, Glucose Enzyme Electrodes. Ann. N.Y. Acad. Sci 102(29), doi: 10.1111/j (1962).

[b5] Sternberg R., Barrau M.-B., Gangiotti L. & Thévenot D. R. Study and development of multilayer needle-type enzyme-based glucose microsensors. Biosens. Bioelectron 4(1), 27–40 (1989). 10.1016/0265-928x(89)80032-x 2751717

[b6] Koudelka M., Gernet S. & Rooij N. F. D., Planar amperometric enzyme-based glucose microelectrode. Sens. Actuators, B 2(18), 157–165 (1989).

[b7] Gorton L., Bremle G., Csöregi E., Pettersson J. & Persson B. Amperometric glucose sensors based on immobilized glucose-oxidizing enzymes and chemically modified electrodes. Anal. Chim. Acta 249(1), 43–54 (1991).

[b8] Malitesta C., Palmisano F., Torsi L. & Zambonin P. J. Glucose fast-response amperometric sensor based on glucose oxidase immobilized in an electropolymerized poly(o-phenylenediamine) film. J. Anal. Chem. 62(24), 2735–2740 (1990). 10.1021/ac00223a016 2096737

[b9] Zhang Y. & Wilson G. S. *In vitro* and *in vivo* evaluation of oxygen effects on a glucose oxidase based implantable glucose sensor. Anal. Chim. Acta 281(3), 513–520 (1993).

[b10] Liu S. & Ju H. Reagentless glucose biosensor based on direct electron transfer of glucose oxidase immobilized on colloidal gold modified carbon paste electrode. Biosens. Bioelectron 19(3), 177–183 (2003). 14611752 10.1016/s0956-5663(03)00172-6

[b11] Zhang J., Hodge W., Hutnick C. & Wang X. Noninvasive Diagnostic Devices for Diabetes through Measuring Tear Glucose. J Diabetes Sci Technol 5(1), 166–172 (2011). 21303640 10.1177/193229681100500123 PMC3045221

[b12] Daum K. M. & Hill R. M. Human tear glucose. e. Invest Ophthalmol Vis Sci 22(4), 509–514 (1982). 7199517

[b13] Panchbha A. S. Correlation of Salivary Glucose Level with Blood Glucose Level in Diabetes Mellitus. J. Oral Maxillofac. Surg. 3(3), doi: 10.5037/jomr.2012.3303 (2012). PMC3886087 24422015

[b14] Moyer J., Wilson D., Finkelshtein I., Wong B. & Potts R. Correlation between sweat glucose and blood glucose in subjects with diabetes. Diabetes Technol. Ther. 14(5), 398–402 (2012 May). 22376082 10.1089/dia.2011.0262

[b15] Sen D. K. & Sarin G. S. Tear glucose levels in normal people and in diabetic patients. Br J Ophthalmol 64, 693–695 (1980). 7426593 10.1136/bjo.64.9.693 PMC1043796

[b16] Kumar S., Padmashree S. & Jayalekshmi R. Correlation of salivary glucose, blood glucose and oral candidal carriage in the saliva of type 2 diabetics: A case-control study. Contemp Clin Dent 5(3), 312–317 (2014). 25191065 10.4103/0976-237X.137925 PMC4147805

[b17] Jonathan C. . Nonostrcturing Platinum Nanoparticles on Multilayered Graphene Petal Nanosheets for Electrochemical Biosensing. Adv. Funct. Mater 22, 3399–3405 (2012).

[b18] Meng L. . Hydrogen Microexplosion synthesis of platnium nanoparticles/nitrogen doped graphene nanoscrolls as new amperometric glucose biosensor. Electrochim. Acta, 330–337 (2015), doi: 10.1016/j.electacta.2014.11.180.

[b19] Shang L., Zhao F. & Zeng B. Highly dispersive hollow PfAg alloy nanoparticles mdified ionic liquid functionalized graphene nanoribbons for electrochemial sensing of nifedipine. Electrochim. Acta 0013(4686), 330–336 (2015).

[b20] Moozarm Niaa P., Menga W. P., Lorestania F., Mahmoudianb M. & Aliasa Y. Electrodeposition of copper oxide/polypyrrole/reduced graphene oxide as a nonenzymatic glucose biosensor. Sens. Actuators, B 209, 100–108 (2015).

[b21] Shang L., Zhao F. & Zeng B. Sensetive valtammetric determination of vanillin with an AuPd nanoparticles-graphene composite modified electrode. Food Chem. 151, 53–57 (2014). 24423501 10.1016/j.foodchem.2013.11.044

[b22] Yuana M. . Bimetallic PdCu nanoparticle decorated three-dimentional graphene hydrogel for non-enzyymatic amperometric glucose sensor. Sens. Actuators, B 190, 707–714 (2014).

[b23] Li M. , Bo X., Mu Z., Zhang Y. & Guo L. Electrodeposition of nickel oxide and platinum nanoparticles on electrochemically reduced graphene oxide film as a nonenzymatic glucose sensor. Sens. Actuators, B, 192, 261–268 (2014).

[b24] Lia Z., Xiea C., Wanga J., Mengb A. & Zhanga F. Direct electrochemistry of cholestrole oxidase immobilized on chitosan-graphene and chholestrole sensing. Sens. Actuators, B 208, 505–511 (2015).

[b25] Fanga Y., Zhangb D., Guoa Y., Guoa Y. & Chenb Q. Simple one-pot preparation of chitosan-reduced graphene oxide-Au nanoparticles hybrids for glucose sensing. Sens. Actuators, B 221, 265–272 (2015).

[b26] Hwaa K. Y. & Subramania B. Synthesis of zinc oxide nanoparticles on graphene–carbon nanotube hybrid for glucose biosensor applications. Biosens. Bioelectron 62, 127–133 (2014). 24997365 10.1016/j.bios.2014.06.023

[b27] Zhang B., He Y., Liu B. & Tang D. Nickel-functionalized reduced graphene oxide with polyaniline for non-enzymatic glucose sensing. Mikrochim. Acta 182(3), 625–631 (2015).

[b28] Shahriary L. & Athawale A. A. Electrochemical deposition of silver/silver oxide on reduced graphene oxide for glucose sensing. J. Solid State Electrochem. 19(8), 2255–2263 (2015).

[b29] Ye Y. . A novel reduction approach to fabricate quantum-sized SnO2-conjugated reduced graphene oxide nanocomposites as non-enzymatic glucose sensors. PCCP 201(19), doi: 10.1039/C4CP04418E, (2014). 24699526

[b30] Geim A. K. Graphene: Status and Prospects. Science 324, 1530–1534 (2009). 19541989 10.1126/science.1158877

[b31] Geim A. K. & Novoselov K. S. The rise of graphene. Nat. Mater 6, 183–191 (2007). 17330084 10.1038/nmat1849

[b32] Martinez A., Fuse K. & Yamashita S. Mechanical exfoliation of graphene for the passive mode-locking of fiber lasers. Appl. Phys. Lett, 1786–1791, doi: 10.1063/1.3641419 (2011).

[b33] Chang Y. M., Kim H., Lee J. & Song Y. Multilayered graphene efficiently formed by mechanical exfoliation for nonlinear saturable absorbers in fiber mode-locked lasers. Appl. Phys. Lett, 203106, doi: 10.1063/1.3521257 (2010).

[b34] Mattevi C., Kim C. & Chhowalla M. A review of chemical vapour deposition of graphene on copper. J. Mater. Chem. doi: 10.1039/c0jm02126a (2001).

[b35] El-Kady M. F., Strong V., Dubin S. & Kaner B. Laser Scribing of High-Performance and Flexible Graphene-Based Electrochemical Capacitors. Science, doi: 10.1126/science.1216744 (2012). 22422977

[b36] El-Kady M. F. & Kaner B. Scalable fabrication of high-power graphene micro-supercapacitors for flexible and on-chip energy storage. Nat. Commun. doi: 10.1038/ncomms2446 (2012). 23403576

[b37] Jiang Liu. . Enhanced non-enzymatic glucose sensing based on copper nanoparticles decorated nitrogen-doped graphene. Biosens. Bioelectron 54, 273–278 (2014). 24287416 10.1016/j.bios.2013.11.005

[b38] Khalid B., Meng Q. H. & Cao Q. H. A non-enzymatic thermally reduced Cu nanoparticle based graphene-resorcinol benzaldehyde glucose sensor. Mater. Res. Innovations 19(2), 91–96 (2015).

[b39] Luo J., Jiang S., Zhang H., Jiang J. & Liu X. A Novel non-enzymatic glucose sensor based on cu nano particle modified graphene sheets electrode. Anal. Chim. Acta, doi: 10.1016/j.aca.2011.10.025 (2012). 22122930

[b40] Mallesha M. . Direct electrochemical non-enzymatic assay of glucose using functionalized graphene. J. Solid State Electrochem 16(8), 2675–2681 (2012).

[b41] Lin J. . Laser-induced porous graphene films from commercial polymers. Nat. Commun. doi: 10.1038/ncomms6714 (2014). PMC4264682 25493446

[b42] Ferrari C. A. Raman spectroscopy of graphene and graphite: Disorder, electron–phonon coupling, doping and nonadiabatic effects. Solid State Commun., 47–57 (2007), doi: 10.1016/j.ssc.2007.03.052.

[b43] Nemanich R. j. & Solin S. A. & Si P. First- and second-order Raman scattering from finite-size crystals of graphite., Phys. Rev. B, 20(2) (1979).

[b44] Pimenta M. A. . Studying disorder in graphite-based systems by Raman spectroscopy. PCCP, 1276–1291 (2007). 17347700 10.1039/b613962k

[b45] Thermo Fisher Scientific Inc, Thermo XPS Scientific- XPS knowledge base. Technical report. (2013) Available at: http://xpssimplified.com/periodictable.php. (Accessed: 20th March 2016).

[b46] Hsua Y. L. . Synthesis of CuO/graphene nanocomposites for nonenzymatic electrochemical glucose biosensor applications. Electrochimica Acta 82, 152–157 (2012).

[b47] Li Y. & Li Y. High performance enzyme free glucose sensor based on the graphene-cuo nanocomposites. Int. J. Electrochem. Sci., 6332–6342 (2013).

[b48] Zhou D. . Facile synthesis of monodisperse porous cu2o nanospheres on reduced graphene oxide for non-enzymatic amperometric glucose sensing. Electrochim. Acta 115, 103–108 (2014).

[b49] Grujicic D. & Pesic B. Electrodepostion of copper: the nucleation mechanism. Electrochim. Acta 47(18), 2901–2912 (2002).

[b50] Yao H., Shum A. J., Cowan M., Lähdesmäki I. & Parviz B. A. A contact lens with embedded sensor for monitoring tear glucose level. Biosens. Bioelectron. *2011 Mar 15;26(7):3290–6* 26(7), 3290–6 (2011). 21257302 10.1016/j.bios.2010.12.042 PMC3043144

[b51] Yamaguchi M. & Kano M. Y., Noninvasively measuring blood glucose using saliva. IEEE Eng Med Biol Mag, 17(3), 59–63 (2002). 9604702 10.1109/51.677170

[b52] Tehrani F., Reiner L. & Bavarian B. Rapid Prototyping of a High Sensitivity Graphene Based Glucose Sensor Strip, PLoS ONE, 10(12), doi: 10.1371/journal.pone.0145036 (2015). PMC4682964 26678700

[b53] Haghighi B., Karimi B., Tavahodi M. & Behzadneia H., Fabrication of a nonenzymatic glucose sensor using Pd-nanoparticles decorated ionic liquid derived fibrillated mesoporous carbon. Mater Sci Eng 219–224 (2015), doi: 10.1016/j.msec.2015.03.045. 25953561

[b54] Yanga J., Zhangb W. & Gunasekaran S. An amperometric non-enzymatic glucose sensor by electrodepositing copper nanocubes onto vertically well-aligned multi-walled carbon nanotube arrays. Biosens. Bioelectron, 279–284 (2010), doi: 10.1016/j.bios.2010.06.014. 20615684

[b55] Luo J., Jiang S., Zhang H., Jiang J. & Liu X. A novel non-enzymatic glucose sensor based on Cu nanoparticle modified graphene sheets electrode. Anal. Chim. Acta 709(4), 47–53 (2012). 22122930 10.1016/j.aca.2011.10.025

[b56] Dharaa K., Stanleyb J., Ta T., Bipin G. N. & Babu S. T. G. Pt-CuO nanoparticles decorated reduced graphene oxide for the fabrication of highly sensitive non-enzymatic disposable glucose sensor. Sens. Actuators, B 195, 197–205 (2014).

[b57] Claussen J. C. . Nanostructuring Platinum Nanoparticles on Multilayered Graphene Petal Nanosheets for Electrochemical Biosensing. Adv. Funct. Mater. 22(16), 3399–3405 (2012).

[b58] Su L. . Colorimetric detection of urine glucose based ZnFe2O4 magnetic nanoparticles. Anal. Chem. 84(13), 5753–8 (2012). 22702236 10.1021/ac300939z

